# Associations of Triglyceride-Glucose Index and Its Derivatives with Hyperuricemia Risk: A Cohort Study in Chinese General Population

**DOI:** 10.1155/2020/3214716

**Published:** 2020-09-17

**Authors:** Qing Gu, Xue Hu, Jian Meng, Jun Ge, Sui Jun Wang, Xing Zhen Liu

**Affiliations:** ^1^Department of Endocrinology, Shidong Hospital, University of Shanghai for Science and Technology, Shanghai, China; ^2^Hangzhou Aeronautical Sanatorium for Special Service of China Air Force, Hangzhou, China

## Abstract

**Background:**

Identification and intervention of insulin resistance may be beneficial to the prevention of hyperuricemia (HUA) and its related diseases. Thus, we conducted this longitudinal study to examine the relation of triglyceride-glucose index (TyG), a simple noninsulin-based IR assessment tool, and its derivatives with the risk of HUA.

**Methods:**

A total of 42,387 adults who received routine health screening and were free of HUA were included for the longitudinal analyses. TyG, body mass index (BMI), waist circumference (WC), and waist-to-height ratio (WtHR) were calculated through anthropometric and biochemical indicators. Associations of TyG, TyG-BMI, TyG-WC, and TyG-WHtR with HUA risk were estimated using Cox regression analyses.

**Results:**

The incident cases of HUA occurred in 4,230 subjects during the 138,163 person-years of observation, and the crude incidence rate of HUA was 30.6 per 1000 person-years. After multivariate adjustment, we observed an increased risk for incident HUA for the upper TyG and its derivatives' tercile. The HRs of TyG were greater than that of its components in both sexes. Compared with TyG, TyG-related parameters only had higher HRs in women but not in men.

**Conclusions:**

TyG and its integration with obesity indicators have the potential to help risk stratification and prevention of HUA, especially among women.

## 1. Introduction

Hyperuricemia (HUA) has gradually become a public health burden due to its widespread prevalence and clinical implications. HUA can not only cause gout and chronic kidney diseases but has also been shown to play an important role in contributing to the development of hypertension, diabetes, and cardiovascular diseases [1, 2]. The kidney is the most important organ for regulating urate levels, which is mainly assisted by various urate transporters distributed in the renal tubules [3]. These urate transporters are all proteins coded by genes, which can be affected by genetic and environmental factors, and their interactions [4, 5].

Insulin is not only the most important hormone that regulates blood glucose levels but has also been shown to be an endogenous regulator of urate in the kidney by influencing the actions of some urate transporters [6]. When insulin resistance (IR) appears, its compensatory hyperinsulinemia may reduce urate excretion by affecting the expression of urate transporters, which contributes to the development and progression of HUA [7]. Therefore, timely identification and intervention of IR could be beneficial to the prevention of HUA and its related diseases.

Due to the close relationship between IR and glycolipid metabolism, triglyceride-glucose index (TyG) was proposed as a simple surrogate of IR by integrating fasting plasma glucose (FPG) and triglycerides (TG) [8]. Subsequent studies have demonstrated the effectiveness of TyG in assessing IR and its related diseases [9, 10]. Furthermore, when TyG combines some adiposity indicators such as body mass index (BMI), waist circumference (WC), and waist-to-height ratio (WtHR), its efficiency in evaluating IR may be improved [11, 12].

Although our previous and some other cross-sectional studies have discussed the correlation between TyG and HUA [13, 14], there are currently no published studies focusing on the longitudinal association of TyG and its derivatives with HUA risk. This retrospective cohort study therefore sets out to evaluate the association of TyG, TyG-BMI, TyG-WC, and TyG-WHtR with the HUA risk. It is hoped that the present study could filter out some simple and effective tools for the stratified prevention of HUA and its related diseases.

## 2. Methods

### 2.1. Study Population

This study was a retrospective cohort study based on the database of individuals who received routine health screening between January 2013 and July 2019 in China. We adopt the method of open cohort study to obtain longitudinal data and enroll the ones who had at least 2 years of continuous data in this study.

The exclusion criteria were pregnant or lactating, with HUA/gout, renal dysfunction, urinary tract infection, or other serious diseases, and taking antihypertensive or lipid-lowering drugs. Finally, a total of 42,387 adults without HUA were analyzed in this study.

### 2.2. Data Collection

The demographic information was collected by the physician. Height and weight were measured by electronic scales. WC and hip circumference (HC) were measured by a well-trained nurse with a soft tape measure. Systolic and diastolic blood pressures (SBP/DBP) and heart rate (HR) were measured 3 times after at least 5 minutes of rest using an automatic blood pressure monitor (HEM-1000, OMRON). The blood samples of subjects were collected after a minimum of 8 h of overnight fasting. Serum levels of FPG, urate, total cholesterol (TC), TG, low-density lipoprotein cholesterol (LDLc), high-density lipoprotein cholesterol (HDLc), and serum creatinine (Scr) were measured by a biochemical autoanalyzer. Abdominal ultrasonography was undertaken by clinical radiologists using a 3.5 MHz probe.

### 2.3. Definitions

HUA diagnosis for men and postmenopausal women was serum urate ≥420 *μ*mol/L and was ≥360 *μ*mol/L in premenopausal women, or receiving urate lowering therapies. The incidence density rate of HUA was calculated by dividing HUA incident by person-years and expressed per 1000 person-years. BMI was calculated as weight divided by the square of height; WC divided by the HP was waist-to-hip ratio (WHR); WC divided by height provided the WHtR; estimated glomerular filtration ratio (eGFR) was calculated using a modified Chinese equation based on insulin clearance [15]. TyG = Ln [fasting TG (mg/dL) ∗ FPG (mg/dL)/2] [8].

### 2.4. Statistical Analysis

Statistical analysis was performed using SPSS 18.0 (SPSS Inc.). Data are reported as means ± SD or percent. Proportions were compared by chi-squared test and means by independent sample *t*-test. Gardner–Altman plots were produced using estimation statistics for data visualization. A Cox proportional hazards regression model was carried out to analyze the hazard ratio (HR) and 95% confidence intervals (CIs) for HUA. TyG and its derivatives were divided into three tertiles and the lowest tertile was used as a reference. The fully adjusted covariates included baseline age, gender, smoking status, overweight/obesity, hypertension, nonalcoholic fatty liver, eGFR, and urate. All probability values were two-tailed and a *p* value < 0.05 was considered to indicate statistical significance.

## 3. Results

At baseline, the mean age of the 42,387 study population was 43.1 ± 12.3 years, and 43.7% subjects were women. The mean FPG, TG, and urate were 5.56 ± 1.13 mmol/L, 1.42 ± 1.18 mmol/L, and 318.9 ± 61.6 *μ*mol/L, respectively. The mean value of TyG, TyG-BMI, TyG-WC, and TyG-WHtR were 8.55 ± 0.62, 198.9 ± 35.9, 673.4 ± 116.5, and 4.06 ± 0.65. The baseline characteristics of subjects according to TyG tertiles are shown in Table 1. Compared with subjects with the lowest tertile of TyG (T1), those with the top tertile of TyG (T3) were more likely to be men, older, fatter, and had an unfavourable metabolic risk factors (all *p* < 0.001).

**Figure 1:**
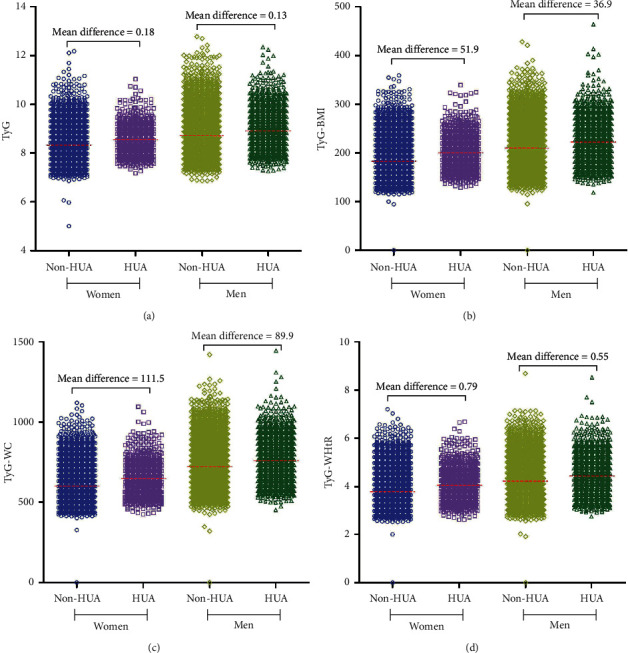
Gardner–Altman plots for TyG and its derivatives according to the presence and absence of HUA. The raw data of TyG and its derivatives are shown on the left axis, and the red line represents its mean value; the mean difference of variables between individual presence and absence of HUA are shown above the raw data; HUA, hyperuricemia; TyG, triglyceride and glucose index; BMI, body mass index; WC, waist circumference; WHtR, waist-to-height ratio.

During the 138,163 person-years of observation, 4,230 incident cases of HUA were identified, and the crude incidence rate of HUA was 30.6 per 1000 person-years. The crude incidence rate of HUA increased from 15.2 per 1000 person-years in the lowest tertile of TyG to 48.3 per 1000 person-years in the top tertile of TyG and showed the similar growth trend in the tertiles of TyG-related parameters (Table 2). In both genders, individuals who developed HUA had significantly higher baseline values of TyG, TyG-BMI, TyG-WC, and TyG-WHtR, and the mean difference among women is greater than that of men (Figure 1).

**Figure 2:**
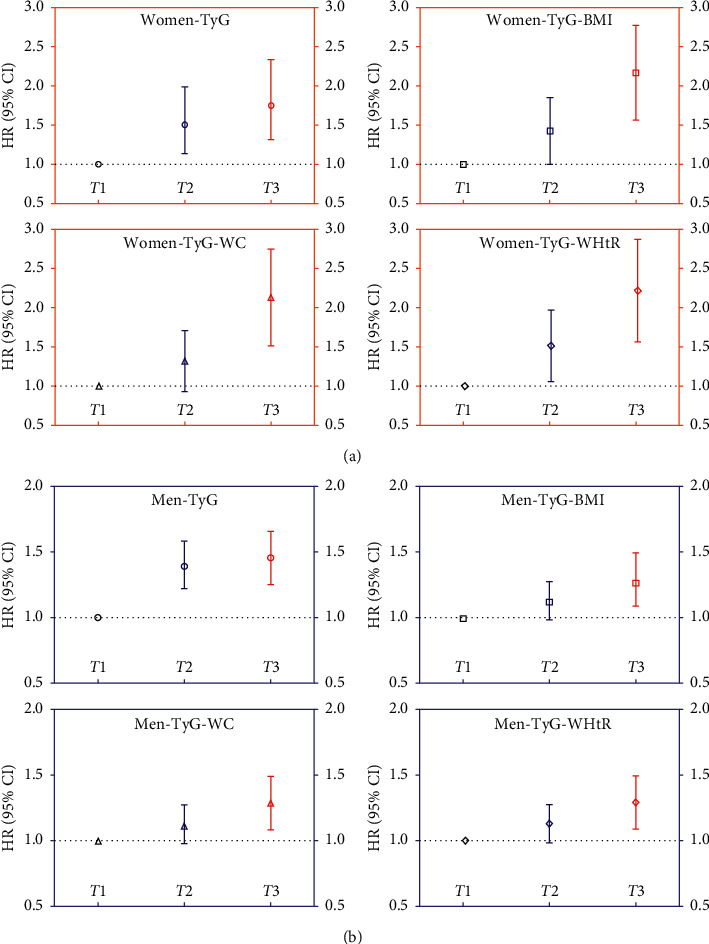
Hazard ratios and 95% confidence intervals for the incidence of hyperuricemia in the tertiles of TyG and its derivatives. The multivariate adjusted HR are adjusted for baseline age, smoking status, overweight/obesity, hypertension, nonalcoholic fatty liver, eGFR, and urate; HR, hazard ratios; CI, confidence intervals; (*T*) tertile; TyG, triglyceride and glucose index; BMI, body mass index; WC, waist circumference; WHtR, waist-to-height ratio.

As shown in Figure 2, after adjusting the potential covariates, the HRs for HUA were higher for TyG than that for its components in both sexes. The HRs for HUA were the highest for TyG-WHtR at 2.122 (95% CI 1.562–2.883) (*p* < 0.001) for the topmost tertile in women; this was followed by TyG-BMI [2.053 (95% CI 1.520–2.773), *p* < 0.001], TyG-WC [2.031 (95% CI 1.506–2.740), *p* < 0.001], and TyG [1.753 (95% CI 1.314–2.337), *p* < 0.001]. In contrast, TyG had the highest HRs for HUA [1.440 (95% CI 1.254–1.654), *p* < 0.001] in men; this was followed by TyG-BMI [1.271 (95% CI 1.099–1.469), *p* < 0.001], TyG-WHtR [1.263 (95% CI 1.091–1.463), *p* < 0.001], and TyG-WC [1.259 (95% CI 1.088–1.457), *p* < 0.001].

## 4. Discussion

In this large-scale retrospective cohort study, we explored the longitudinal association of TyG with the HUA risk. Furthermore, we also investigated whether the combination of TyG and some anthropometric indicators can improve this association. Our data revealed that TyG was more associated with HUA risk than its components (TG and FPG). Additionally, TyG-related parameters were also significantly associated with HUA risk in both sexes, and TyG integrates obesity indicators can strengthen the association in women but not in men.

With lifestyle changes and obesity epidemic, HUA has become a global health issue [16]. In this study, 10% of subjects were observed developed HUA, and the crude incidence rate of HUA was 30.6 per 1000 person-years. This result was consistent with previous studies. Two studies based on the Chinese population reported the incidence of HUA were 12.1% and 15.6%, respectively [17, 18]. Another study based on the Japanese observed the incidence rate of HUA was 31.7 per 1000 person-years [19]. Considering the potential centrality of HUA in metabolic diseases, although the vast majority of HUA are asymptomatic, the prevention and management of HUA deserves further attention.

The regulation of urinary urate excretion is very complicated and requires the participation of various urate transporters, which are involved in the secretion (ABCG2) and reabsorption (URAT1 and GLUT9) of urate [20]. The dysfunctional variants in these genes have been shown to have stronger effects on the risk of HUA [21]. Stiburkova et al. showed that the ABCG2 allelic variants (p.Q141 K) are highly associated with early onset HUA which has a familial background [22]. In addition to genetic factors, insulin can also affect the level of urate by interacting with these urate transporters. In normal individuals, insulin can reduce the urinary excretion of sodium and urate [23]. When IR occurs, acute physiological hyperinsulinemia can stimulate urate reabsorption by regulating the expression of URAT1 and ABCG2 [24].

Furthermore, IR/hyperinsulinemia can not only reduce the excretion of urate but also contribute to its overproduction. Generally, moderate levels of insulin suppress lipolysis efficiently in normal humans, but this effect will weaken in individuals with IR/hyperinsulinemia [25]. With the enhancement of lipolysis, excessive free fatty acids are generated and then lead to dyslipidemia and the subsequent overgeneration of urate [26].

Even though the close relationship between HUA and IR has been well demonstrated, using this theory to guide the prevention and management of HUA in clinical practice is still one step away and that is IR assessment. The hyperinsulinemic-euglycemic clamp (HEC) is considered the gold standard to assess IR [27]. However, due to its invasiveness, complexity, and time consuming properties, HEC is difficult to use in clinical practice. As an alternative strategy, the homeostasis model assessment for the IR (HOMA-IR) index is much simpler than HEC [28]. However, insulin testing still needs to consider the cost and repeatability issues, especially in primary health institutions [29]. The proposal of TyG quickly attracted attention attributed to it and derived from faster and inexpensive biochemical markers. Several studies have demonstrated that TyG maintains good consistency with HEC and HOMA-IR [9]. Therefore, TyG provides more options for the evaluation of IR and the prevention of IR-related diseases in clinical practice.

Although there have been more studies on the connection between TyG and the risk of hypertension or diabetes, studies on the association of TyG with the HUA risk are still rare. There are currently only two cross-sectional studies exploring the relationship between TyG and HUA [13, 14]. In this cohort study, TyG shows significant association with increased HUA risk, which complemented previous research by determining the causative effect of TyG and the underlying IR on the development of HUA [30].

The relationship between FPG, one of the components of TyG, and urate is more complicated. Many studies have demonstrated that an invert “U-shape” with a threshold of FPG was existed for urate [31]. Furthermore, there is a gender difference at the inflection point values of FPG. Whitehead et al. reported the inflection point values were 9.0 mmol/L in women and 7.0 mmol/L in men [32]. So, the changes of FPG and urate are more likely to be consistent among women, which may partially explain the value of HR for TyG and its related parameters in women were higher than that of men.

Obesity plays a vital role in the pathophysiology of IR [33], so TyG combined with obesity indicators should be able to strengthen the role of TyG theoretically. Several studies have demonstrated the superiority of TyG combined with obesity indicators [11, 12], and our recent study also shows that TyG-BMI was more associated with prehypertension in nonobese adults than TyG [34]. In the present study, the associations of TyG combined with obesity indicators with the HUA risk were prone to change in different genders. Overall, TyG-related parameters only outperformed TyG in women but not in men. These results may be explained by the sex differences in fat distribution, glycolipid metabolism, and urate metabolism [35]. It is worth noting that TyG-WHtR had the highest value of HR in women. This result may be related to WHtR and can reflect more information about visceral obesity than the other three traditional obesity indicators [36].

The main strength of this retrospective cohort study was a large sample size, which could provide more statistical power. However, some of the limitations of this study should also be noted. First, the observation time was relatively shorter. Second, the socio-economic information and diet structure were not adjusted, which may cause some potential bias. Third, our findings seem to be ethnic dependent, which should be further verified in other ethnic populations.

In conclusion, TyG, a noninsulin-based IR index that is based on routine biochemical tests, was significantly associated with HUA risk in both sexes. Additionally, TyG combined with some obesity indicators (especially WHtR) may strengthen its association with the HUA risk in women but not in men. Thus, TyG and its related parameters have the potential to be used as risk grading tools for HUA prevention in clinical practice. However, additional studies with longer observation times will be needed to confirm this finding.

## Figures and Tables

**Table 1:** Baseline characteristics of subjects grouped according to the tertiles of TyG.

Variable	Total	TyG			*p* value^*∗*^
		T1 (<8.24)	T2 (8.24–8.75)	T3 (<8.75)	
No., n	42,387	14129	14127	14131	—
Women (%)	43.7	63.7	42.5	24.8	<0.001
Age, y	43.1 ± 12.3	38.4 ± 11.2	43.4 ± 12.3	47.4 ± 11.7	<0.001
SBP (mmHg)	121.2 ± 16.7	115.4 ± 14.9	121.0 ± 16.2	127.2 ± 16.8	<0.001
DBP (mmHg)	73.6 ± 11.3	69.4 ± 10.1	73.4 ± 10.9	77.9 ± 11.2	<0.001
HR (bmp)	79.9 ± 12.2	80.2 ± 12.4	79.6 ± 12.2	79.9 ± 12.0	<0.001
BMI (kg/m^2^)	23.2 ± 3.1	21.6 ± 2.6	23.1 ± 2.9	24.8 ± 2.9	<0.001
WC (cm)	78.4 ± 9.7	72.7 ± 7.9	78.2 ± 8.9	84.3 ± 8.7	<0.001
WHR	0.84 ± 0.07	0.80 ± 0.06	0.84 ± 0.06	0.88 ± 0.06	<0.001
WHtR	0.47 ± 0.05	0.44 ± 0.04	0.47 ± 0.05	0.50 ± 0.05	<0.001
Urate (*μ*mol/L)	318.9 ± 61.6	292.6 ± 60.0	318.9 ± 59.3	345.3 ± 53.5	<0.001
FPG (mmol/L)	5.56 ± 1.13	5.15 ± 0.46	5.42 ± 0.59	6.11 ± 1.66	<0.001
TC (mmol/L)	4.76 ± 0.88	4.42 ± 0.77	4.75 ± 0.81	5.12 ± 0.93	<0.001
TG (mmol/L)	1.42 ± 1.18	0.70 ± 0.15	1.15 ± 0.19	2.41 ± 1.59	<0.001
HDLc (mmol/L)	1.56 ± 0.36	1.74 ± 0.34	1.58 ± 0.33	1.40 ± 0.32	<0.001
LDLc (mmol/L)	2.50 ± 0.77	2.29 ± 0.62	2.56 ± 0.68	2.61 ± 0.91	<0.001
eGFR (mL/min/1.73 m^2^)	79.4 ± 20.3	83.1 ± 18.9	80.6 ± 21.1	78.2 ± 19.8	<0.001
TyG	8.55 ± 0.62	7.93 ± 0.24	8.49 ± 0.14	9.24 ± 0.46	<0.001
TyG-BMI	198.9 ± 35.9	171.2 ± 22.0	196.0 ± 25.6	229.6 ± 31.6	<0.001
TyG-WC	673.4 ± 116.5	577.1 ± 68.7	664.2 ± 78.1	779.0 ± 97.0	<0.001
TyG-WHtR	4.06 ± 0.65	3.51 ± 0.38	4.00 ± 0.43	4.65 ± 0.55	<0.001

*T*, tertile; SBP, systolic blood pressure; DBP, diastolic blood pressure; HR, heart rate; BMI, body mass index; WC, waist circumference; WHR, waist-to-hip ratio; WHtR, waist height ratio; FPG, fasting plasma glucose; TC, total cholesterol; TG, triglyceride; HDLc, high-density lipoprotein cholesterol; LDLc, low-density lipoprotein cholesterol; eGFR, estimated glomerular filtration rate; TG, triglyceride; the asterisk means *pvalue* for the difference of variables between *T*1 and *T*3.

**Table 2:** The crude incidence rate of hyperuricemia categorised by the tertiles of TyG and its derivatives.

	Person-years	Incident HUA	Crude incidence rate
Total	138,163	4,230	30.6
*TyG*			
T1	46,690	710	15.2
T2	45,782	1,314	28.7
T3	45,691	2,206	48.3
*TyG-BMI*			
T1	46,247	654	14.1
T2	46,147	1,272	27.6
T3	45,769	2,304	50.3
*TyG-WC*			
T1	46,280	568	12.3
T2	46,043	1,309	28.4
T3	45,840	2,353	51.3
*TyG-WHtR*			
T1	46,162	688	14.9
T2	46,100	1,307	28.4
T3	45,901	2,235	48.7

HUA, hyperuricemia; *T*, tertile; TG, triglyceride; FPG, fasting plasma glucose; TyG, triglyceride and glucose index; BMI, body mass index; WC, waist circumference; WHtR, waist-to-height ratio; the crude incident rate is expressed per 1000 person-years.

## Data Availability

The datasets used and/or analyzed during the current study are available from the corresponding author on reasonable request.
